# Involvement of Raphe Nuclei in Depressive-like Behaviors and Short-Term Memory in an Animal Model of Parkinson’s Disease

**DOI:** 10.1021/acschemneuro.5c00987

**Published:** 2026-06-25

**Authors:** Daniele C. Ramos, Jeane C. F. Vieira, Leonardo C. Souza, Mariza Bortolanza, Elaine Del Bel, Roberto Andreatini, Maria A. B. F. Vital

**Affiliations:** † Department of Pharmacology, Federal University of Parana, Curitiba, PR 81531-980, Brazil; ‡ Department of Morphology, Physiology and Pathology, Dentistry School, University of São Paulo, Ribeirão Preto, SP 14040-904 Brazil

**Keywords:** Parkinson’s disease, depression, memory, raphe nuclei, rotenone

## Abstract

Parkinson’s disease (PD) is a neurodegenerative disease characterized by motor symptoms and nonmotor disturbances such as fatigue, cognitive impairment, constipation, anxiety, and depression. The dopaminergic neurodegeneration is a hallmark of PD; however, the serotonergic system also presents alterations. Dorsal raphe nucleus (DRN) and median raphe nucleus (MNR) neurons are responsible for the central biosynthesis of serotonin (5-HT). We aimed to investigate whether the rotenone model would affect serotonergic neurons of the DRN and MRN and reduce 5-HT levels in target structures related to depressive-like behavior and short-term memory. Adult male Wistar rats were treated for 10 days with rotenone or sunflower oil. One day after the last rotenone injection, animals exhibited hypolocomotion in the open field. Furthermore, rotenone impaired the short-term memory assessed in the social recognition test performed 22 days after the last injection. In the forced swim test, performed on day 28, we observed depressive-like behavior, indicated by an increase in immobility time in the rotenone group. Immunohistochemical analysis revealed a reduction of serotonergic neurons in the MRN on the 28th day after the last administration of rotenone. Moreover, the ventrolateral part of DRN presented a decrease of serotonergic neurons. Neurochemical quantification revealed a reduction of serotonin levels in the prefrontal cortex and striatum, and a reduction of dopamine and noradrenaline levels in the prefrontal cortex, striatum, and amygdala. Overall, the results revealed that the development of behavioral impairments induced by systemic rotenone is involved with MRN, and likewise 5-HT, other neurotransmitters such as dopamine and noradrenaline may play a role in nonmotor disorders in the animal model of PD.

## Graphical abstract

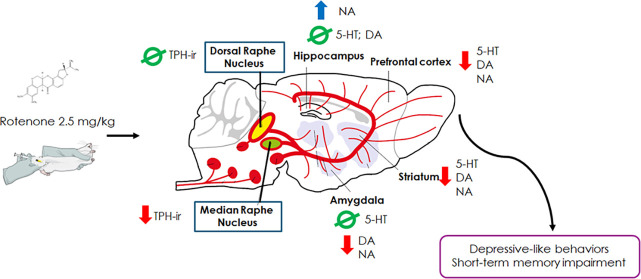

## 1 Introduction

Parkinson’s disease (PD) is a neurodegenerative disease characterized by motor signs; however, patients also develop nonmotor disturbances such as fatigue, cognitive impairment, constipation, anxiety, and depression.^1–3^ PD has a complex progression, and the neurodegeneration occurs beyond the dopaminergic system, affecting other brain areas including the locus coeruleus and raphe nuclei.^4,5^ According to Braak’s staging of PD hypothesis, neuropathological alterations in neurons of the raphe nuclei arise from stage 2, which precede the alterations in substantia nigra pars compacta (SNpc) during stage 3.^6^ The loss of serotonergic neurons prior to the dopaminergic might explains, in part, the occurrence of depression in the premotor phase of DP.^7,8^ In the central nervous system (CNS), serotonin-containing neurons are located, mainly, in the dorsal raphe nucleus (DRN), and to a lesser extent in the median raphe nucleus (MRN).^9,10^ Both nuclei share many projection areas, however neurons from the rostral region of DRN send their axons to the forebrain and the basal ganglia, while midrostrocaudal and caudal regions send projections to other forebrain structures, such as the amygdala, the hippocampus, septum, and locus coeruleus.^10–13^ Additionally, the MRN projects to medial structures, including the paraventricular thalamic nuclei, hippocampus, caudate putamen, olfactory bulb, medial prefrontal and anterior cingulate cortices.^10,11^ The serotonergic system plays a role in several physiological processes, e.g., neuroendocrine function, regulation of gastrointestinal motility, cognition, and emotion.^10,14,15^ Furthermore, dysfunction of serotonergic transmission has been implicated in some nonmotor disturbances of PD, such as depression, anxiety, and mild cognitive impairment, which have a great impact on patients’ quality of life.^16–19^

A number of studies have demonstrated that alterations in raphe nuclei are associated with the pathogenesis of depression and anxiety in PD patients.^20–24^ Post-mortem investigations have found that the serotonin-synthesizing neurons loss in the dorsal raphe nucleus is more severe in depressed than nondepressed PD patients,^20^ and neurons in the median raphe are more affected in PD patients when compared to healthy volunteers.^25^ Furthermore, some studies with PD patients have suggested a reduction in tryptophan hydroxylase levels (TPH), as well as in serotonin reuptake transporter (SERT) levels in structures that receive raphe projections, such as the striatum, limbic and cortical areas.^26^

Animal studies of PD seek to relate the nigrostriatal neurodegeneration and its impact on the serotonergic system. The results demonstrated that rats with intranigral lesions induced by toxins such as 6-OHDA, MPTP and rotenone had reduced serotonin (5-HT) hippocampal levels. In addition, levels of 5-HT in the hippocampus and levels of DA in the striatum correlated negatively with the depressive-like behavior of the lesioned animals.^27,28^ These studies sought to quantify 5-HT levels in the hippocampus and were limited to analyze the extent of death of dopaminergic neurons in the SNpc. However, few studies have evaluated the effect of the nigrostriatal pathway damage on serotonergic neurons of the raphe nuclei. In this framework it was demonstrated that in the 6-OHDA-lesioned rats that intrastriatal lesion caused a loss of dopaminergic neurons in the dorsal raphe nucleus.^29^ However, no decrease in serotonergic neurons was observed when compared to the control group. Conversely, it was showed that the systemic administration of rotenone led to a lower number of TPH-positive cells and a down-regulation of 5-HT1A receptors in the dorsal raphe nucleus.^30^

Considering the inconsistency of results and the relevance of serotonergic system dysfunction in nonmotor disturbances of PD, we aimed to investigate whether rotenone exposure is able to affect serotonin-producing neurons in the dorsal and median raphe nuclei, and to evaluate whether serotonin levels are reduced in projection structures related to depressive- and anxiety-like behavior and short-term memory (Figure 1).

**Figure 1:**
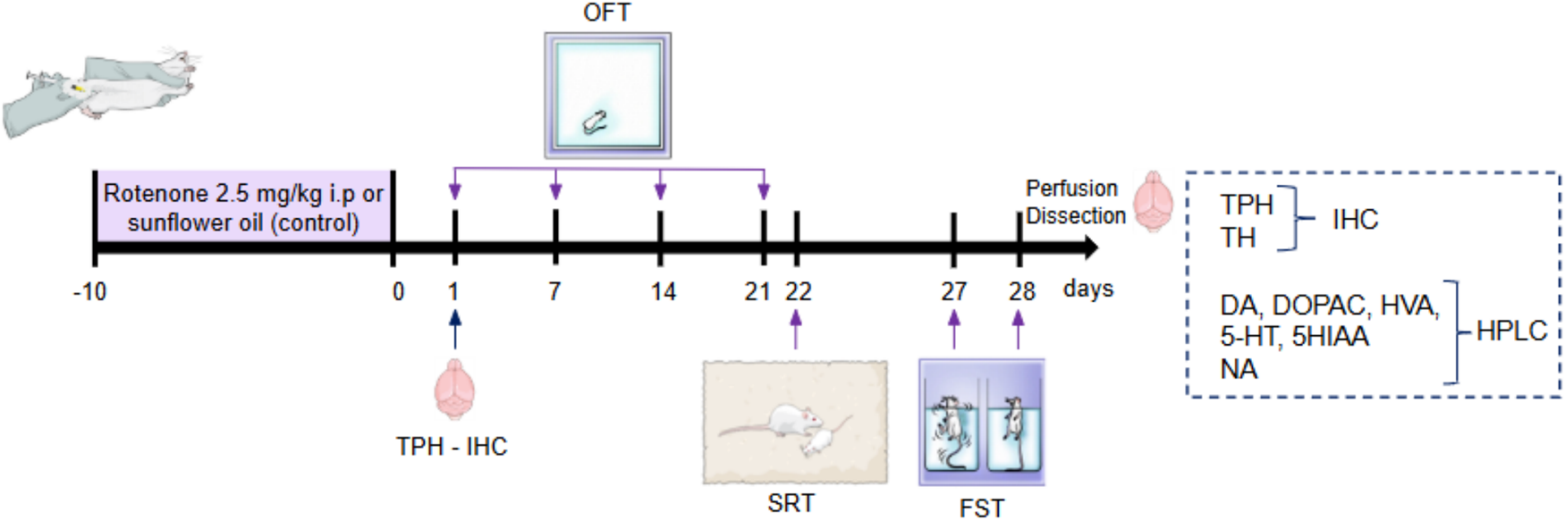
Experimental design. Rats received a single intraperitoneal (i.p) injection of rotenone at 2.5 mg/kg or 1.0 mL/kg of vehicle (sunflower oil) once a day for 10 days. Twenty-4 h after the last day of treatment (day 1), both groups were subjected to the open field test, and then repeated on days 7, 14, and 21. A subset of rats was perfused for TPH immunohistochemical analysis at time-point 24 h after the open field test. On day 22 rats underwent the social recognition test. The training session of the forced swim test was performed on day 27, and the test session on day 28. Immediately after the last behavioral test, animals were either perfused for immunohistochemical analysis, or had their brain structures dissected for neurochemical quantification. OFT: open field test; SRT: social recognition test; FST: forced swim test; DA: dopamine; DOPAC: 3,4-dihydroxyphenylacetic acid; HVA: Homovanillic acid; 5-HT: serotonin; 5HIAA: 5-hydroxy indoleacetic acid; NA: noradrenaline; HPLC: High Performance Liquid Chromatography; TPH: tryptophan hydroxylase; TH: tyrosine hydroxylase; IHC: immunohistochemistry.

## 2 Results

### 2.1 Rotenone Led to Hypolocomotion in the Open Field 24 h after the Last Day of Administration

Three parameters were analyzed in the open field at four different time points: 24 h, 7, 14, and 21 days after the last rotenone injection. As can be seen in Figure 2A, the rotenone group demonstrated a significant difference in the distance traveled (*p* < 0.0001) compared to the control group 24 h after the last rotenone administration, as indicated by the condition [*F*_(1, 15)_ = 9.327, *p* = 0.0080] and interaction factors [*F*_(3, 45)_ = 6.014, *p* = 0.0016] but not time factor [*F*_(3, 45)_ = 2.077, *p* = 0.1166]. Mean speed was significantly decreased in the rotenone group at the time point 24 h compared to the control group (*p* < 0.0001; Figure 2B), as indicated by the condition [*F*_(1, 15)_ = 9.267, *p* = 0.0082] and interaction factors [*F*_(3, 45)_ = 6.072,*p* = 0.0015] but not time factor [*F*_(3, 45)_ = 2.084, *p* = 0.1157]. Similarly, line crossings were significantly decreased in the rotenone group at the time point 24 h compared to the control group (*p* < 0.0001; Figure 2C), as indicated by the condition [*F*_(1, 15)_ = 6.972, *p* = 0.0185] and interaction factors [*F*_(3, 45)_ = 6.218, *p* = 0.0013] but not time factor [*F*_(3, 45)_ = 0.9839, *p* = 0.4088]. Furthermore, at the time-points 7, 14 and 21days, no significant difference was observed between control and rotenone groups in locomotor function analyzed in the three parameters distance traveled, mean speed, and line crossings.

**Figure 2:**
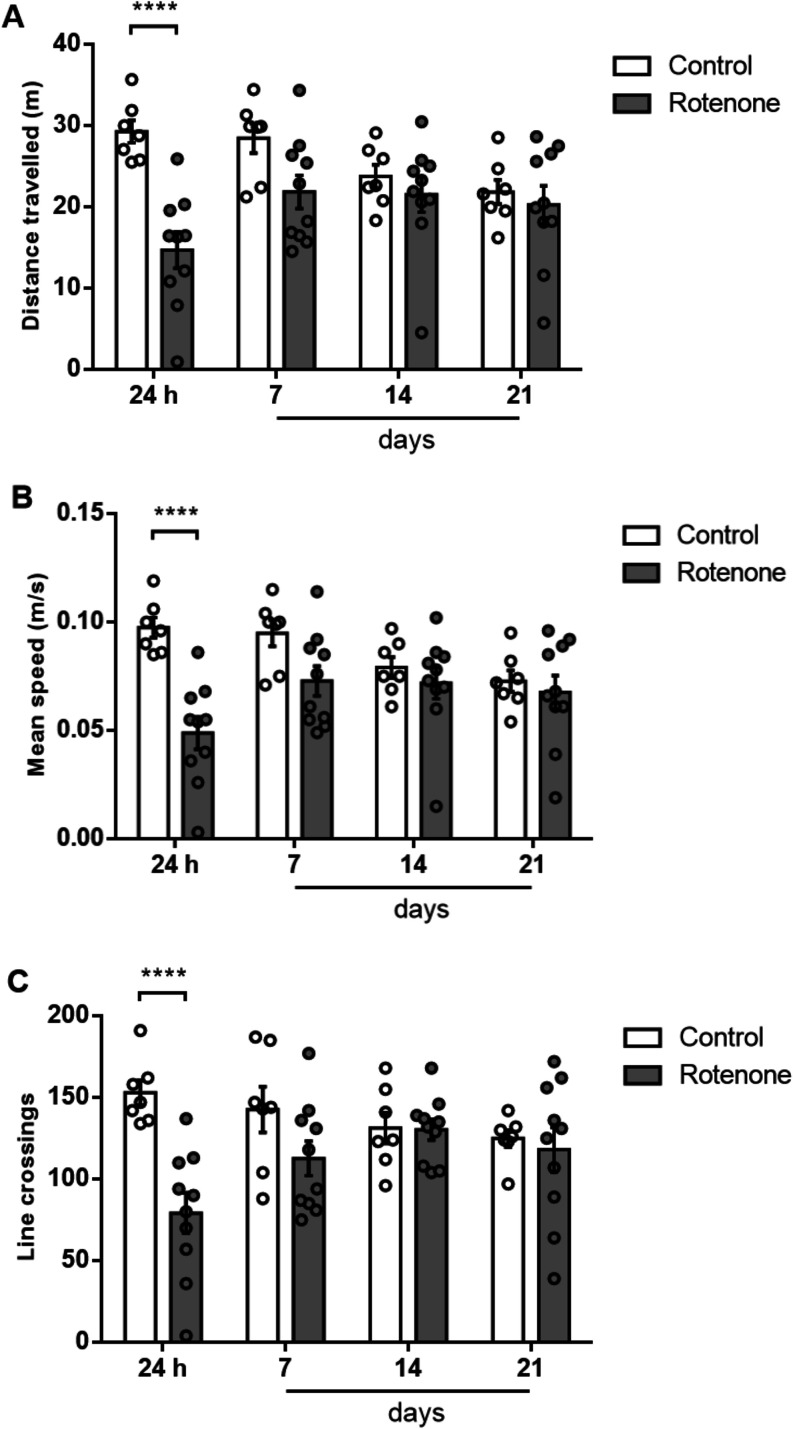
Locomotor behavior assessed in the open field. (A) Distance traveled. (B) Mean speed. (C) Line crossings. The data were obtained 24 h, 7, 14, and 21 days after the last rotenone injection (2.5 mg/kg, i.p., once a day for 10 days). The values are expressed as mean ± SEM (*n* = 7–10/group) and analyzed by two-way ANOVA followed by Bonferroni post hoc test). ****p < 0.0001, when compared to the control group.

### 2.2 Rotenone Administration Impaired the Short-term Social Memory

The duration of investigation in the second encounter was significantly shorter compared with the first presentation in the control group (*p* < 0.05; Figure 3A). Conversely, no significant difference was observed between the first and second presentations in the rotenone group. As can be seen in Figure 3B, the rotenone group showed a significant increase in the ratio of investigation duration (RID) [*t*(21) = 2.828, *p* = 0.0101] compared with the control group. An increased RID indicates that the investigation time did not decrease when the same juvenile rat was re-exposed, suggesting an impairment in the social recognition memory of the rotenone-treated rats.

**Figure 3:**
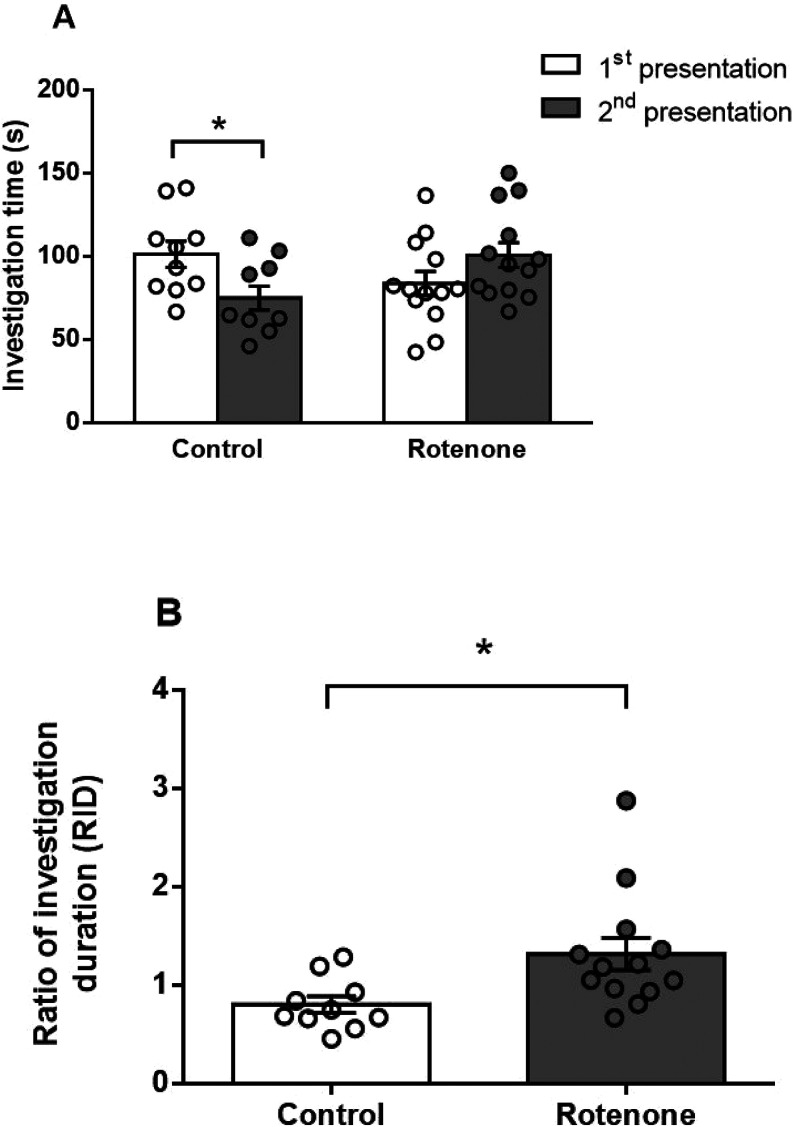
Effects of the administration of rotenone or vehicle on the social recognition memory of rats evaluated 22 days after the last injection (2.5 mg/kg, ip, once a day for 10 days). (A) Investigation time. (B) Ratio of investigation duration. Data are expressed as the mean ± SEM (*n* = 10–13/group); * *p* < 0.05, (A) in comparison with the first presentation (two-way ANOVA followed by Bonferroni post hoc test); (B) in comparison with the control group (two-tailed unpaired Student’s *t*-test).

**Figure 4:**
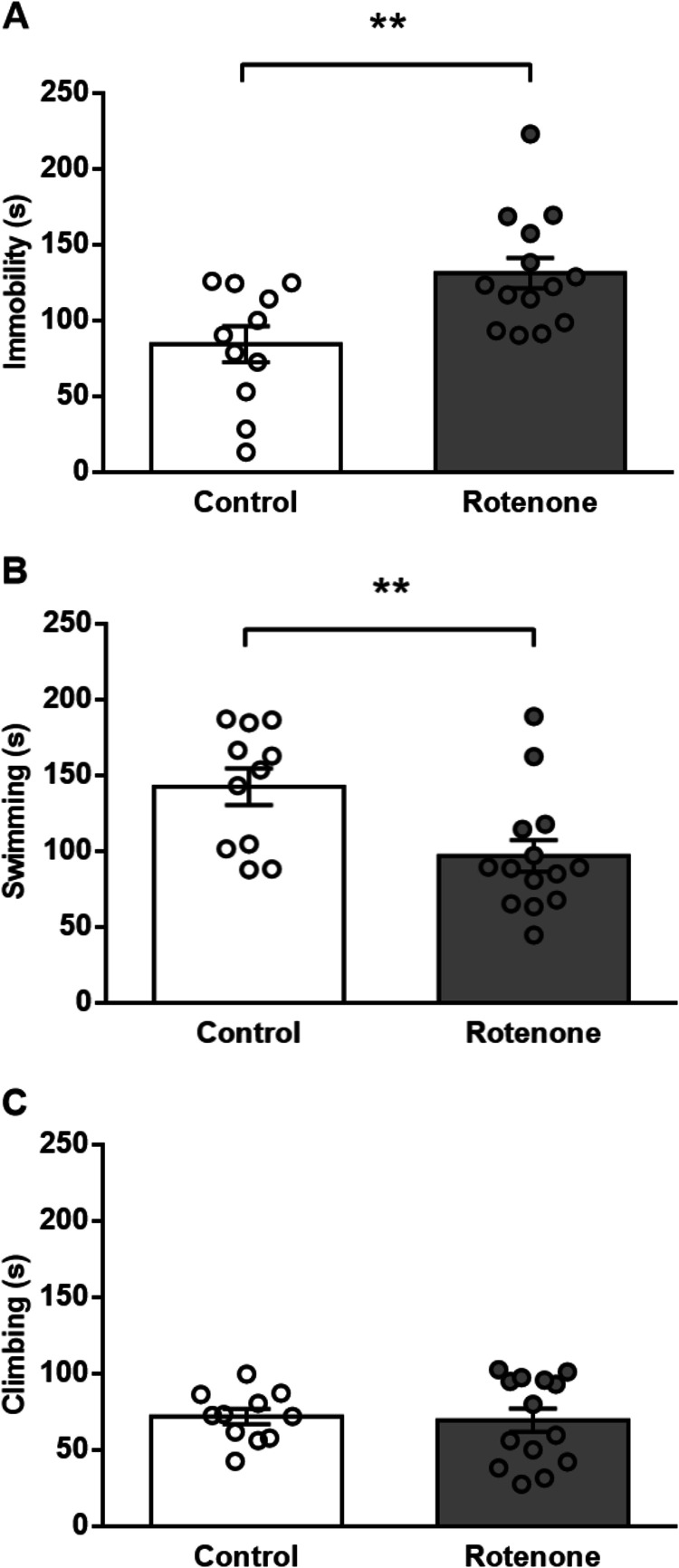
Depressive-like behavior assessed 29 days after the last rotenone exposure (2.5 mg/kg, i.p., once a day for 10 days). (A) Immobility. (B) Swimming. (C) Climbing. Values are expressed as mean ± SEM (*n* = 11–14/group). **p < 0.01, compared with the control group (two-tailed unpaired Student’s *t*-test).

### 2.3 Rotenone Exposure Induced Depressive-like Behavior

A significant increase in immobility time was observed in the rotenone group compared with the control group [*t*_(23)_ = 3.035; *p* = 0.0059; Figure 4A]. In addition, a significant decrease in swimming time was observed in the rotenone animals [*t*_(23)_ = 2.875; *p* = 0.0086; Figure 4B], compared with the control ones. In contrast, no significant difference in climbing time was observed between both groups [*t*_(23)_ = 0.4543; *p* = 0.6539; Figure 4C].

**Figure 5:**
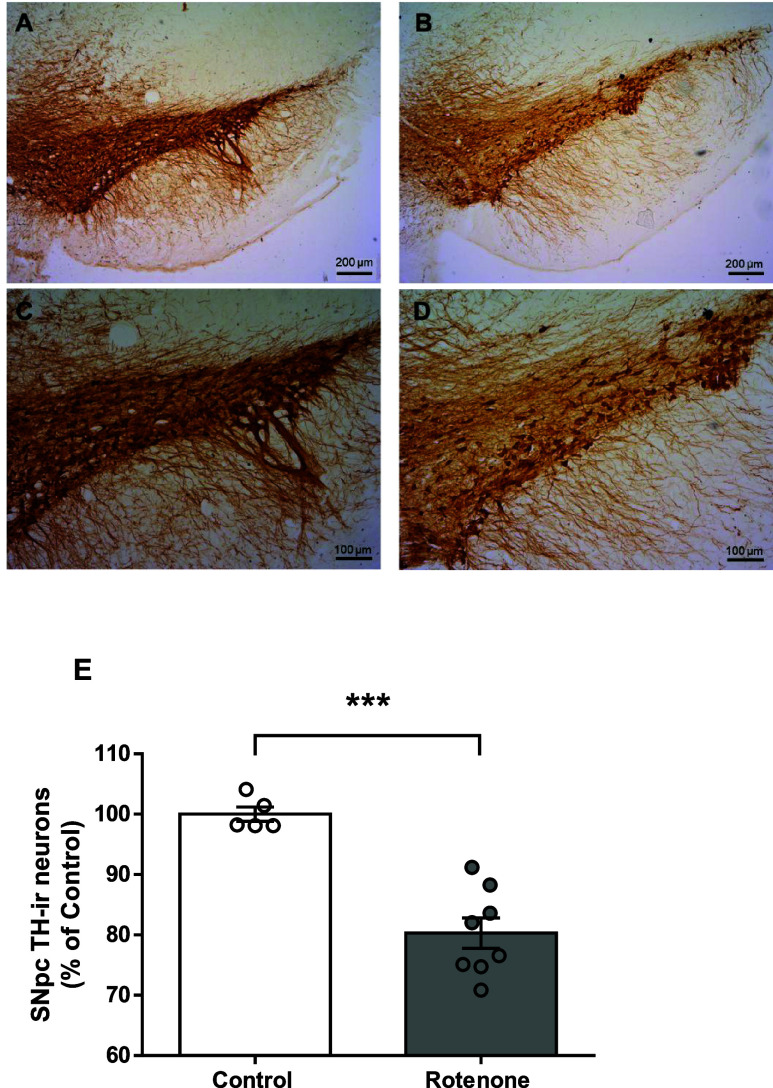
Immunohistochemical analysis of the SNpc 29 days after the last rotenone exposure (2.5 mg/kg, i.p., once a day for 10 days). (A) Photomicrographs of representative sections of tyrosine hydroxylase immunoreactive (TH-ir) neurons in the following groups: (A) control (5× magnification), (B) rotenone (5× magnification), (C) control (10×), (D) rotenone (10×). (E) Quantification of TH-ir neurons in the SNpc. The values are expressed as a percentage of the control group (*n* = 5–8/group). ***p < 0.001, compared with control (two-tailed unpaired Student’s *t*-test). TH-ir: tyrosine hydroxylase immunoreactive.

### 2.4 Rotenone Administration Led to A Partial Lesion in SNpc

The death of DA neurons in the SNpc induced by the systemic administration of rotenone was assessed by the quantification of TH-ir. As shown in Figure 5E, the rotenone group exhibited a significant reduction in TH-ir neurons, in comparison with the control group [*t*_(11)_ = 5.830; *p* = 0.0001]. Thus, the result of the immunohistochemical analysis, performed on the 28th day after the end of rotenone administration, revealed a 20% decrease in TH-ir neurons in the group treated with the neurotoxin.

### 2.5 Rotenone Administration Affected Only the Serotonergic Neurons in the Ventrolateral Subregion (DRVL)

Immunohistochemical analysis of serotonergic neurons in DRN was performed at two time points: 24 h and 28 days after the last administration of rotenone. In addition, neuronal quantification was performed in different subregions of the DRN at four levels in the rostrocaudal extension, according to the bregma: −7.20; −7.80; −8.04 and −8.52 mm. At the rostral level of DRN, at the −7.20 mm coordinate, one-way ANOVA revealed that systemic administration of rotenone had no effect on the number of TPH-ir neurons. The absence of effect was observed in the dorsal subregion (DRD) [*F*_(2, 13)_ = 2.482; *p* = 0.1222; Figure S1E], and in the ventral subregion (DRV) [*F*_(2, 13)_ = 0.6536; *p* = 0.5365; Figure S1E]. As shown in Figure 6J, at the medial level of DRN (coordinate −7.80 mm), the administration of rotenone did not affect the number of neurons in the DRV subregion [*F*_(2, 14)_ = 0.5083; *p* = 0.6122], and in the DRV subregion [*F*_(2, 14)_ = 0.4332; *p* = 0.6569]. Conversely, in the ventrolateral subregion (DRVL) the administration of rotenone affected the number of TPH-ir neurons [*F*_(2, 14)_ = 5.626; *p* = 0.0161], and the reduction was significant in the time points of 24 h and 28 days, compared to the control group. Further analysis of DRN, at the level of the −8.04 mm coordinate (Figure S1O), there was no effect of rotenone administration on the DRD subregion [*F*_(2, 13)_ = 2.391; *p* = 0.1305], in the DRV subregion [*F*_(2, 13)_ = 1.699; *p* = 0.2210], in the DRVL subregion [*F*_(2, 13)_ = 1.258; *p* = 0.3167], and in the interfascicular part (DRI) [*F*_(2, 13)_ = 0.1278; *p* = 0.8811]. Similarly, at the caudal level of DRN (coordinate −8.52 mm), the administration of rotenone did not affect the number of neurons in the caudal subregion (DRC) [*F*_(2, 10)_ = 1.760; *p* = 0.2213; Figure S1T], and in the DRI subregion [*F*_(2, 10)_ = 0.2517; *p* = 0.7823; Figure S1T].

**Figure 6:**
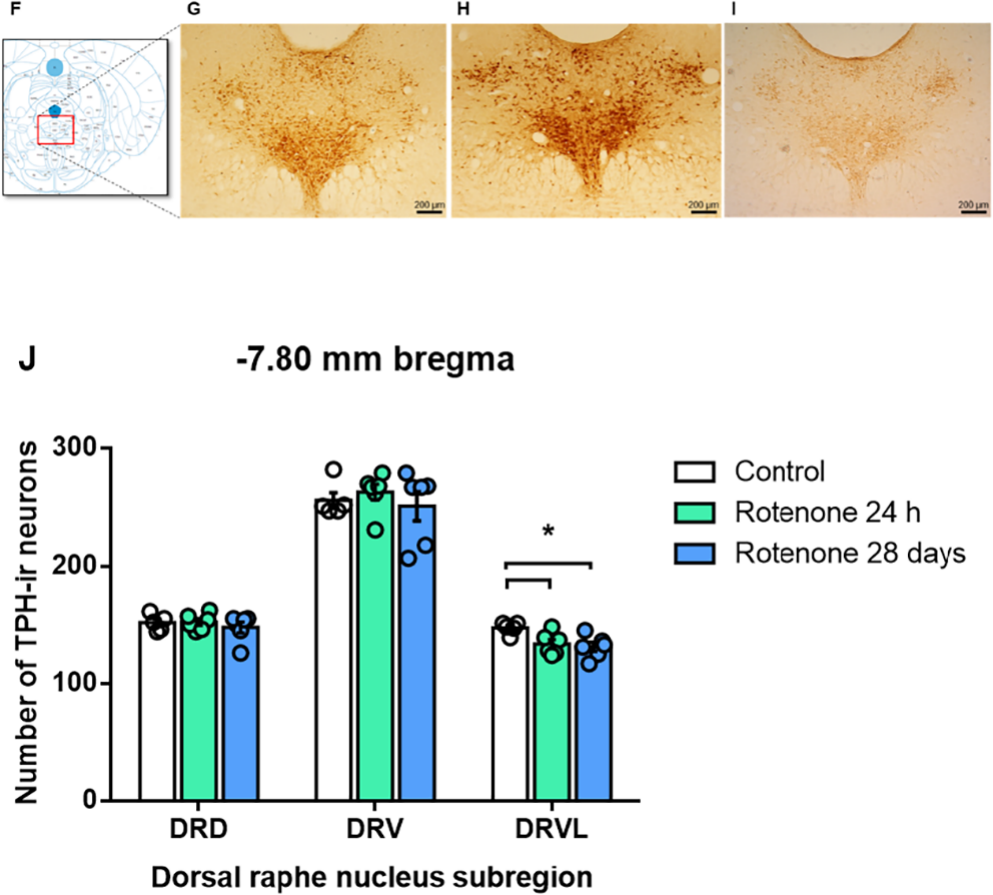
Effect of rotenone administration on serotonergic neurons of DRN labeled for TPH was evaluated 24 h and 28 days after the last rotenone injection (2.5 mg/kg, i.p., once a day for 10 days). (F) Clipping of the Paxinos & Watson atlas indicating the DRN subregions analyzed according to the coordinate −7.80 mm. (G) Representative photomicrography of medial DRN TPH-ir neurons in the control group. (H) Rotenone group 24 h. (I) Rotenone group 28 days. (J) Stereological quantification of the total number of TPH-ir neurons in the DRN subregions for each group. The values are expressed as mean ± SEM (*n* = 5–6/group). *p < 0.05, compared to the control group (one way ANOVA followed by Tukey’s *post hoc* test). DRN: dorsal raphe nucleus; DRD: DRN dorsal part; DRV: DRN ventral part; DRVL: DRN ventrolateral part; TPH-ir: tryptophan hydroxylase immunoreactive.

### 2.6 Rotenone Exposure Led to A Reduction in TPH-ir Neurons in MRN

Immunohistochemical analysis of serotonergic neurons in MRN was also performed 24 h and 28 days after the last administration of rotenone. Stereological quantification of TPH-ir neurons was performed at coordinate −7.80 mm, according to bregma. As shown in Figure 7H, rotenone administration affected the number of TPH-ir neurons [*F*_(2, 14)_ = 5.801; *p* = 0.0146], and the reduction observed was significant only in the rotenone group at the time point 28 days, compared to the control group (*p* < 0.05).

**Figure 7:**
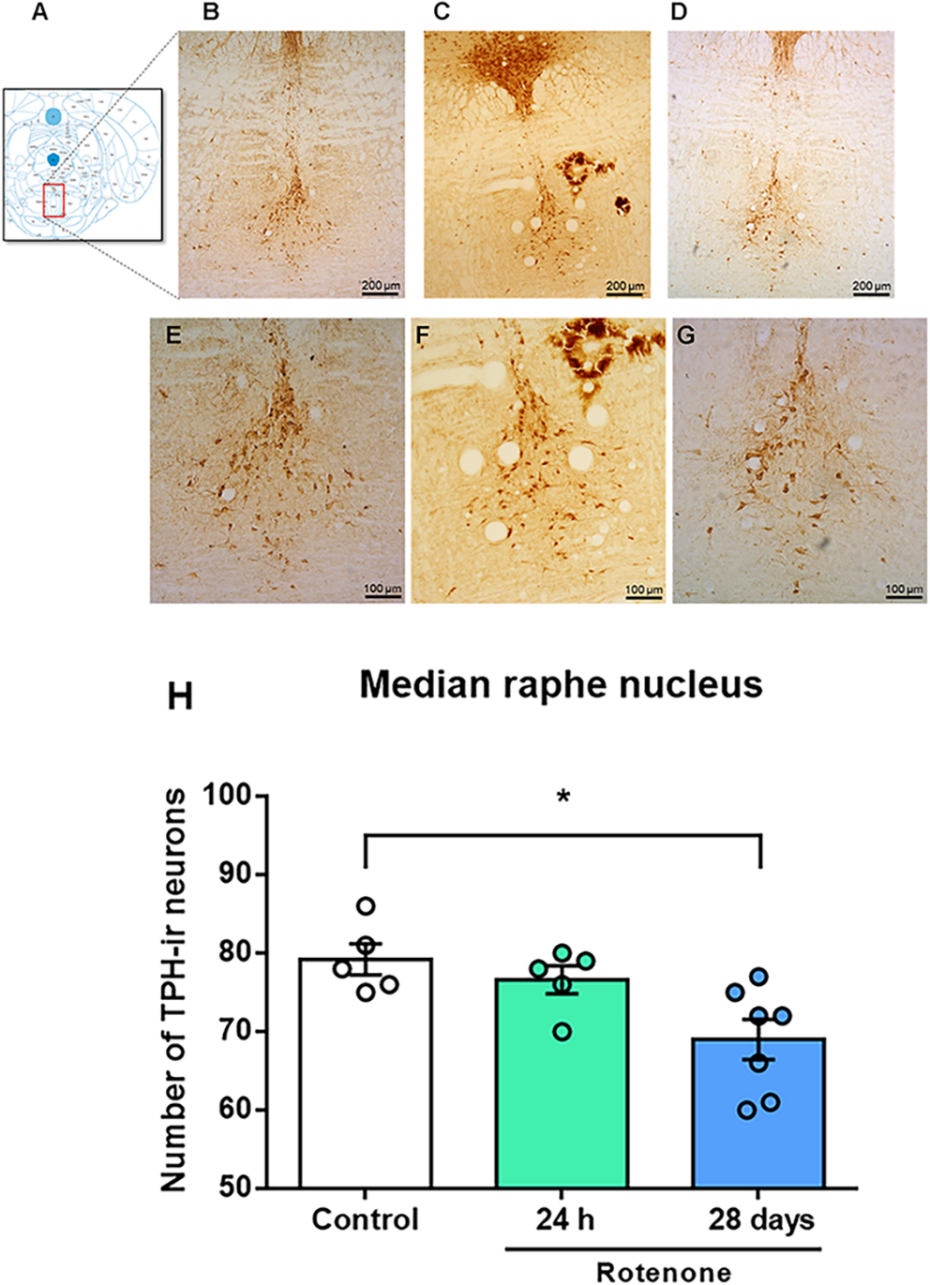
Effect of rotenone administration on serotonergic neurons of MRN labeled for TPH was evaluated 24 h and 28 days after the last rotenone injection (2.5 mg/kg, i.p., once a day for 10 days). (A) Clipping of the Paxinos & Watson atlas indicating the MRN region analyzed according to the coordinate −7.80 mm. (B) Representative photomicrography of MRN TPH-ir neurons in the control group (5× magnification). (C) Rotenone group 24 h (5×). (D) Rotenone group 28 days (5×). (E) Control group (10× magnification). (F) Rotenone group 24 h (10×). (G) Rotenone group 28 days (10×). (H) Stereological quantification of the total number of TPH-ir neurons in MRN for each group. The values are expressed as mean ± SEM (*n* = 5–7/group). * *p* < 0.05, compared to the control group (one way ANOVA followed by Tukey’s *post hoc* test). MRN: median raphe nucleus; TPH-ir: tryptophan hydroxylase immunoreactive.

### 2.7 Rotenone Administration Caused Distinct Effects on Monoamine Levels

Neurochemical analysis of monoamine levels was performed at the end of behavioral procedures, 28 days after the last administration of rotenone. The results showed that 5-HT levels were significantly reduced in the prefrontal cortex [*t*_(16)_ = 2.731; *p* = 0.0148) and in the striatum [*t*_(18)_ = 4.004; *p* = 0.0008) in the rotenone group, when compared to the control. However, no significant differences were found in the 5-HT levels between the groups in the amygdala and hippocampus (Figure 8). Regarding DA levels, the analysis of rotenone group revealed a significant reduction in the prefrontal cortex [*t*_(18)_ = 2.982; *p* = 0.0080), in the striatum [*t*_(18)_ = 6.184; *p* < 0.0001], and in the amygdala [*t*_(18)_ = 6.238; *p* < 0.0001], when compared to the control. Conversely, no significant differences were found in DA levels in the hippocampus between the groups rotenone and control. Furthermore, the results regarding NA levels demonstrated a significant increase in the hippocampus of rotenone groups [*t*_(15)_ = 2.688; *p* = 0.0169], when compared to control. However, the neurochemical analysis of NA revealed a significant reduction in the prefrontal cortex [*t*_(18)_ = 4.291; *p* = 0.0080), in the striatum [*t*_(18)_ = 6.184; *p* < 0.0001], and in the amygdala [*t*_(18)_ = 6.238; *p* < 0.0001], when compared to the control (Figure 8).

**Figure 8:**
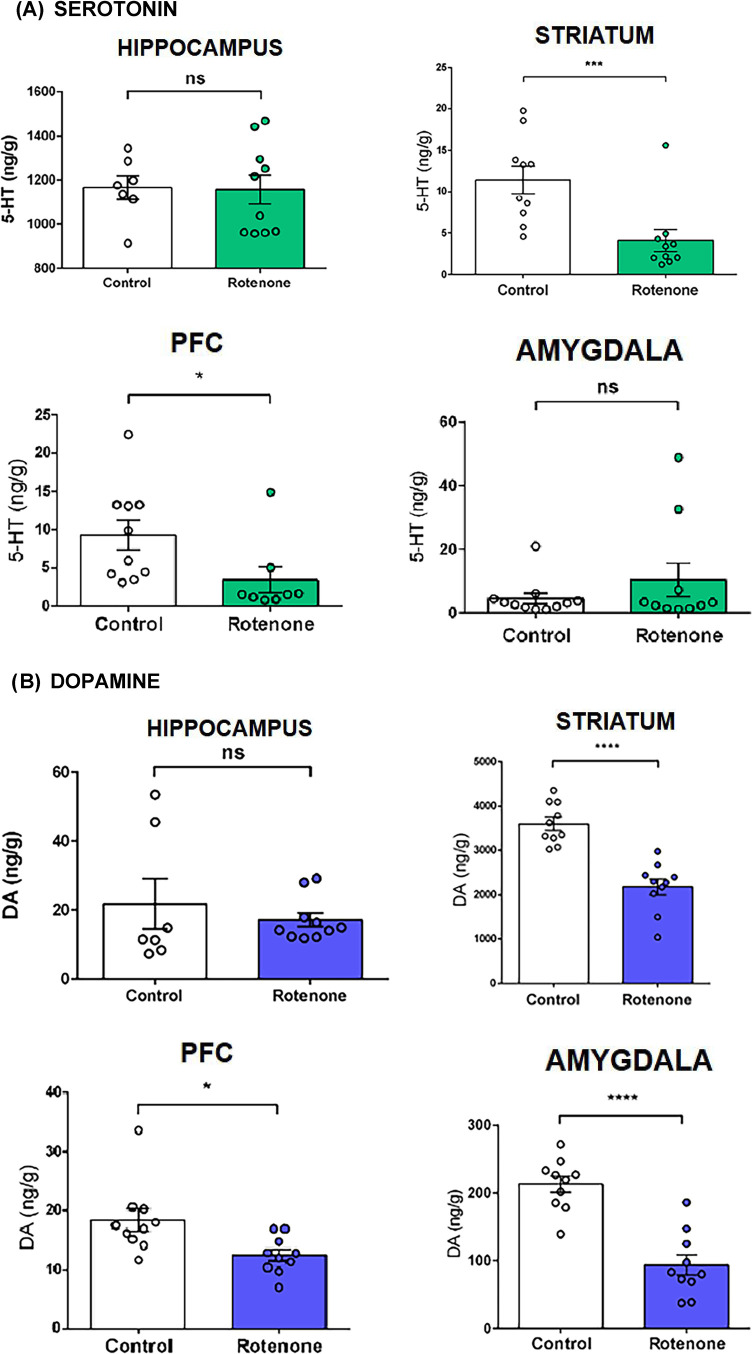

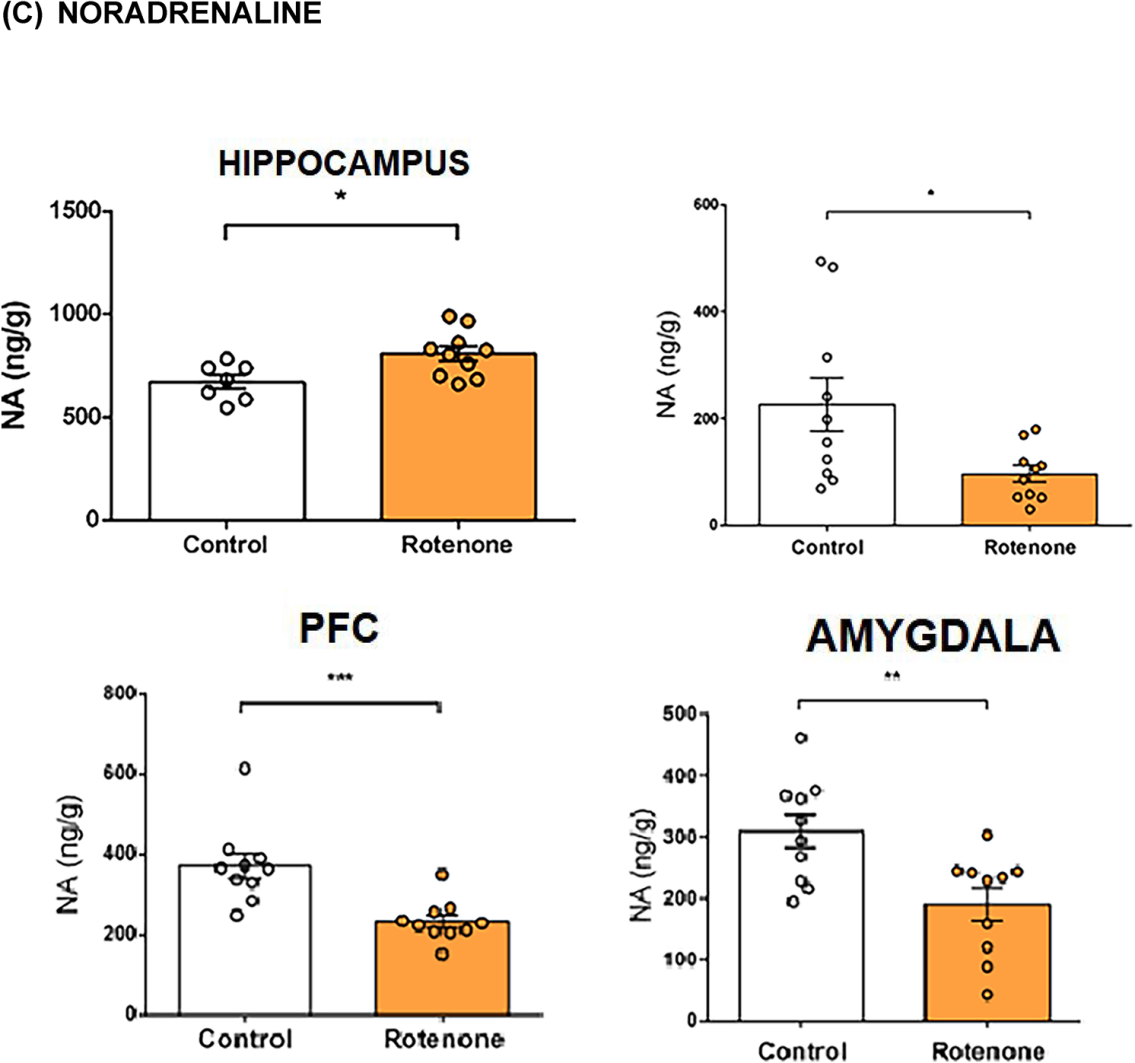
Effect of rotenone administration on neurochemical quantification of (A) 5-HT, (B) DA and (C) NA levels evaluated 28 days after the last injection (2.5 mg/kg, i.p., once a day for 10 days). Quantifications were expressed as ng/g of wet tissue weight. The values are expressed as mean ± SEM (*n* = 7–10/group). *p < 0.05, **p < 0.01, ***p < 0.001, compared to the control group (unpaired two-tailed Student’s *t* test). 5-HT: serotonin, DA: dopamine; NA: noradrenaline.

## 3 Discussion

The present study showed that the rotenone model of PD caused a reduction of the serotonergic neurons in the DRN (DRVL part) and MRN, besides a reduction of 5-HT levels in the striatum and prefrontal cortex (PFC). Moreover, the neurotoxin also reduced the dopaminergic neurons in the SNpc and led to decreased DA levels in the striatum, PFC and amygdala. Likewise, noradrenaline levels were also reduced in the striatum, PFC and amygdala. Probably, these changes contributed to depressive-like behavior and impaired short-term memory exhibited by the animals.

Our results showed that rotenone caused hypolocomotion in the open field test 24 h after the last day of rotenone exposure. In addition, the locomotor parameters analyzed on days 7, 14, 21, and 28 days after the last injection did not demonstrate motor impairment in the rotenone group, similar to control animals. Furthermore, previous studies demonstrated that the rotenone group presented hypolocomotion 24 h after the last exposure to this neurotoxin.^31–34^ In addition, it is known that animals submitted to the same protocol (systemic rotenone 2.5 mg/kg for 10 days) presented a spontaneous motor recovery and this finding can be explained by neuroplasticity events that balance neuronal death.^31^

Depressive-like behavior was assessed in the modified forced swim test. The results demonstrated that rotenone administration led to depressive-like behavior indicated by an increase in immobility time (“behavioral despair”) observed in the rotenone group compared with the control animals. It has been observed that a single intranasal administration of MPTP caused a depressive-like behavior in rats, reflected by an increased immobility time in the forced swim test. Nevertheless, the authors did not observe anhedonic-like behavior in these same animals in the sucrose preference test.^35^ The etiology of depression in PD is complex and may result from changed 5-HT brain chemistry that is related to the central dopaminergic deficiency associated with PD motor signs.^36^ The notion of a DA etiology for depression in PD is not surprising and is somewhat supported by clinical research showing a high association between mood changes and lesions to the basal ganglia.^37^ Thus, observations of pathological features in the SNpc of depressed PD patients, though only trending toward significance, appear relevant and bolster the notion that the nigrostriatal circuit is implicated in the depression of PD.^38^ Even with such importance, there is a lack of studies concerning animal models of PD and depressive-like behaviors in order to elucidate the neurobiology and the interaction between these disorders. Thus, an animal model that can mimic the depression-PD association would be a valuable tool to study this clinical problem. Recent findings support that animal models of PD are able to produce depressive-like behaviors as a comorbidity of PD.^27,39,40^

Additionally, to depressive-like behaviors, the present findings demonstrated a negative effect of the administration of rotenone on the social recognition test in rats. The rotenone group spent as much time investigating the juvenile rat during the second presentation as they did on the first encounter, demonstrating impairment in the ability to recognize the juvenile after a short period of time (30 min). These findings corroborate other studies in which rats with nigrostriatal lesion presented impairment in short-term memory, evaluated in the same paradigm used in this study.^34,35,39^ Studies indicate that MPTP-lesioned rats obtained low scores in the two-way active avoidance task, indicating an impaired learning and memory.^41,42^ In the aforementioned studies, memory impairment seems to be related to the reduction of DA concentration in the prefrontal cortex and striatum, suggesting that the dopaminergic system in these regions plays an important role in memory and learning. However, these processes possibly have the participation of other systems, such as the serotonergic. It has been noted that intracerebroventricular infusion of a serotonergic neurotoxin led to impairment in short-term memory, evaluated in the spontaneous alternation test in the Y-maze, and in working memory evaluated in the 8-arm radial maze. Moreover, the low scores obtained in these tests were associated with the reduction of 5-HT levels in the prefrontal cortex.^43^ Regarding our data obtained in the social recognition test, the short-term memory impairment observed in the rotenone group may be associated with the reduction of 5-HT levels in the prefrontal cortex. Furthermore, the rotenone group showed a reduction in striatal DA levels. Thus, we can suggest that nigrostriatal pathway dysfunction, observed in this study, is involved in short-term memory impairment.

Parkinson’s disease (PD) is more than a movement disorder and patients also develop nonmotor disturbances such as cognitive impairment, anxiety, and depression. In light of this, not only an impaired nigrostriatal pathway but also a dysfunctional serotonergic system is involved in the pathogenesis of the disorders aforementioned. Regarding, anxiety-like behavior, we did not observe this behavioral phenotype, however, it was demonstrated that aged rats (11-month-old) receiving rotenone (1.5 mg/kg/day, s.c., for 6 weeks) spent more time in the periphery during the open field test, suggesting increased level of anxiety.^44^ Thus, it was showed that rotenone administration to rats for 28 days (10 mg/kg p.o.) caused an increase in the number of entries and time spent in closed arms in the elevated plus maze test.^45^ Moreover, a reduction in the number of entries and time spent, as compared to the vehicle-treated control rats in open arms, indicates an increase in anxiety-like behavior in this PD model.

The central 5-HT system comprises 5-HT-producing neurons located in brainstem raphe nuclei, mainly in the dorsal raphe nucleus (DRN) and medial raphe nucleus (MRN).^13^ The DRN is a bilateral, heterogeneous nucleus, located in the ventral periaqueductal gray matter of the midbrain. The neurons of the DRN provide an array of serotonergic innervation throughout the forebrain and represent nearly 50% of the 5-HT neurons in the rat brain.^46–50^ Based on its heterogeneity, the DRN can be divided topographically into subregions, in which neurons receive synaptic input from specific forebrain and brainstem structures and send efferents to forebrain and brainstem structures, such as prefrontal cortex, striatum, amygdala, and ventral hippocampus.^47,48,50^ Therefore, it is important to distinguish the organization of DRN in order to understand the role of serotonin in the development of nonmotor disturbances. Additionally, the MRN, a distinct nucleus in the rostral pons, is the second major source of serotonergic cells and sends efferent projections to the hypothalamus, dorsal hippocampus, and medial septum.^51–53^ In this study, we analyzed the number of TPH-ir neurons in accordance with DRN subregions along the rostrocaudal levels. Our results revealed that rotenone-lesioned rats did not present significant changes in the number of TPH-ir of DRN subregions (dorsal, ventral/ventromedial, ventrolateral, caudal, and interfascicular) along the rostrocaudal axis. Intriguingly, we observed a significant decrease of TPH-ir neurons in the ventrolateral subregion in the middle portion of DRN (−7.80 mm bregma). In addition, this reduction was found in both time-points of 24 h and 28 days after the last rotenone exposure. Indeed, the administration of rotenone led to distinct effects in raphe nuclei. Moreover, our data demonstrated a significant decrease of TPH-ir neurons in the MRN of rotenone-lesioned rats in comparison with the control group, 28 days after the last rotenone injection.

These data corroborate, partially, some studies in different animal models of PD. One study show that intrastriatal 6-OHDA lesion in rats did not reduce the number of serotonergic neurons in the DRN.^29^ In addition, in the A53T transgenic mouse model of PD, accumulations of α-synuclein were detected in the cellular bodies of serotonergic neurons of DRN and MRN. However, no decrease in the number of TPH-ir neurons in both nuclei was observed, although there was a reduction in the 5-HT levels in these same nuclei.^54^ Conversely, our data showed that there was a reduction of TPH-ir neurons in MRN in the rotenone model. In fact, the results related to serotonergic neurons are contradictory. In this regard, it was demonstrated that systemic administration of rotenone (3.0 mg/kg for 14 days) induced a decrease of TPH-ir neurons and a reduced expression of 5-HT in the DRN.^30^ Furthermore, studies in monkeys with a systemic MPTP administration model have shown no differences in the density of TPH-ir neurons in the DRN, and in the somatodendritic morphology of serotonergic neurons, compared to the control.^55^ The presence of α-synuclein aggregates is suggested to be involved in the dysfunction of the serotonergic system, and the death of 5-HT body cells in raphe nuclei. However, the present study did not analyze the presence of these aggregates and this is a limitation of our study. With respect to the face validity of the rotenone model - defined as the phenomenological similarity between the model and the clinical condition - it is noteworthy that numerous studies employing various routes of administration and dosages have demonstrated that rotenone can induce neurodegeneration and α-synuclein accumulation in both the central and enteric nervous systems of rodents.^56–58^ In addition, it is necessary the employment of a more extended rotenone protocol to induce the accumulation of α-synuclein aggregates.^59^

Data obtained from PD patients are slightly contradictory. Evidence suggests that MRN serotonergic neurons are more affected than DRN neurons.^14,25^ In addition, some post-mortem analysis of PD patients revealed α-synuclein aggregates in the cellular bodies of serotonergic neurons labeled for TPH-ir, and these findings were accompanied by neuronal loss in MRN.^60^ Otherwise, other post-mortem studies have demonstrated a greater loss of serotonergic neurons in DRN in depressed PD patients than in nondepressed PD patients.^20^ Some factors can contribute to these conflicting data, such as differences in drug treatments, duration of illness, etc. These factors can be controlled in animal models.

We quantified the 5-HT levels in target structures related to those disorders. Our data revealed that rotenone exposure results in decreased levels of 5-HT in the striatum and prefrontal cortex. Conversely, no significant difference was detected in the hippocampus and amygdala of rotenone-lesioned rats when compared to control. Interestingly, we did not observe a reduction of serotonin neurons labeled for TPH in DRN subregions that send projections to the striatum and prefrontal cortex. Studies have indicated that rostral DRN neurons, in ventral and dorsal subregions, provide 5-HT innervation to the striatum.^46,53,61^ Moreover, ventromedial and caudal DRN subregions send efferents to the hippocampus.^11,46,48,53^ In this case, we can suggest that the deregulation of this serotonergic pathway is not related to the number of serotonergic neurons *per se* but related to a downregulation of 5-HT receptors or the 5-HT transporter (SERT) in the striatum.^62^ Studies suggest that SNpc neurodegeneration may lead to a reduction in the content of striatal 5-HT. Furthermore, it is possible that the alterations, in this case, occur primarily in the axon terminals and then in serotonergic cell bodies.^63^

According to Braak,^6^ noradrenergic neurons are degenerated in the locus coeruleus of PD patients prior to SNpc DA neurons degeneration. Clinical data showed that brain regions that receive noradrenergic innervations had significantly lower [11C]-RTI-32 binding, a marker for both noradrenaline and dopamine transporter, in depressed patients with PD when compared to nondepressed patients with PD.^64^ However, it was reported evidence from both animal and clinical data indicating early dysfunction of noradrenergic transmission in circuits of the central and peripheral nervous systems. Moreover, supported by clinical phenotyping in vivo neuroimaging studies, these authors have suggested the existence of distinct nonmotor endophenotypes of PD.^65^

Regarding the animal models of PD, there is a consensus in the literature that it is considerably difficult to reproduce the nonmotor features, particularly anxiety and depression in rodents.^66,67^ However, there is an amount of evidence confirming that the administration of rotenone reproduces depressive-like behavior, indicating good face validity.^30,31,58,66,68^

It has been found that serotonergic neurons exhibit greater vulnerability to rotenone than nonserotonergic neurons.^69^ In midbrain neuronal cultures, they also demonstrated that rotenone induces microtubule depolymerization in addition to its well-established inhibition of mitochondrial complex I, consistent with earlier observations.^56^ In this line, 5-HT and tryptophan hydroxylase (TPH) expressions in the dorsal raphe were suppressed by rotenone (3 mg/kg for 14 days) injection.^30^

Corroborating the present results, it was demonstrated that administration of rotenone (1.5 mg/kg/day) to rats for 8 days resulted in motor deficits that were subsequently accompanied by depressive-like behaviors.^68^ These behavioral changes were evaluated using standard behavioral paradigms: forced swim test, open field test, sucrose preference test, and social interaction test. This finding was further confirmed by reduced dopamine and serotonin levels in striatum and hippocampus and strengthened the monoaminergic hypothesis that depression in PD involves a complex interaction between 5-HT and DA.

Noradrenaline levels have been relatively understudied in animal models of depression in PD, and the available results are often contradictory.^66^ Our results showed that the content of this neurotransmitter was also reduced in the striatum, PFC, and amygdala. Surprisingly, hippocampal noradrenaline levels were increased. This increase in monoamine levels may represent a compensatory response to the neurotoxin. In this line, increased level of NA was shown in the prefrontal cortex and hippocampus of 6-OHDA lesioned rats while no significant difference was found in rats intracranially injected with other neurotoxins, such as MPTP, rotenone, or LPS.^27,39^ On the other hand, a recently published study showed that the prolonged rotenone administration at a dose of 2 mg/kg/day for 21 days to mice caused a significant reduction in dopamine and noradrenaline hippocampal levels in comparison to the control group in the 22nd day.^70^

Taken together, these results suggest that the discrepancies in noradrenaline levels observed in PD models likely depend on factors such as the type of neurotoxin, dose, duration of administration, and species studied. Ongoing experiments may help clarify these observations.

## 4 Material and Methods

### 4.1 Animals

Male Wistar rats from Universidade Federal do Parana (UFPR) vivarium were used, weighing 280–320 g at the beginning of the experiments. Rats were housed in groups of four in polypropylene cages and maintained in a temperature-controlled room (22 ± 2 °C) on a 12/12 light/dark cycle (lights on at 7 a.m.) with food and water ad libitum. All possible efforts were made to minimize the number of animals used and their discomfort during the experimental procedures. All procedures were approved by the Animal Care and Use Committee of the UFPR (protocol number 1154) and conducted in accordance with the Brazilian regulation (11.794/8 October 2008) and the National Institutes of Health Guidelines for the Care and Use of Laboratory Animals.

### 4.2 Drugs

Rotenone (Sigma, St. Louis, MO) was dissolved in sunflower oil at a final concentration of 2.5 mg/mL. This solution was injected intraperitoneally (i.p) at a dose of 2.5 mg/kg. The control group received the rotenone vehicle (sunflower oil) at 1 mL/kg, i.p.

### 4.3 Experimental Design

Eighty rats were randomly distributed into two groups: control (rotenone vehicle) and rotenone. The rotenone (2.5 mg/kg, i.p.) was administered once a day for 10 consecutive days – protocol used to induce a nigrostriatal lesion.^31,32^ Both groups of rats underwent all the experimental procedures. Motor behavior was assessed by the open field test at different time-points: 1, 7, 14, and 21 days after the last rotenone exposure. A subset of animals was perfused for TPH immunohistochemical analysis at time-point 24 h after the open field test. Short-term memory was evaluated in the social recognition test on 22 day. Finally, animals were tested in the modified forced swim test 27 days (training session) and 28 days (test session) after the last rotenone injection, to assess the depressive-like behavior. At the end of the last test, the rats were intracardially perfused for immunohistochemical analysis. Another subset of animals was anesthetized, and the brains removed for neurochemical procedures (Figure 1).

### 4.4 Behavioral Assessments

#### 4.4.1 Open Field Test

The apparatus consisted of a circular arena (97 cm diameter, 42 cm wall height) into three concentric circles and subdivided into 19 quadrants. Animals were gently placed in the center of the apparatus and allowed to freely explore the area for 5 min. Three motor parameters were recorded: distance traveled (i.e., the distance in meters that the animal traveled during the test); speed of locomotion (i.e., speed of locomotion in meters/second that the animal performed during the test); and line crossings. The open-field arena was cleaned with a 5% water–ethanol solution before behavioral testing to eliminate possible bias caused by odors left by previous rats. Animal movements were monitored and recorded with a video tracking system – ANY-maze (Stoelting Co., IL).

#### 4.4.2 Social Recognition Test

Short-term memory was assessed with the social recognition test described previously.^71,72^ This test is based on the behavior of adult male rats which spend a great amount of time investigating novel juveniles. In contrast, rats re-exposed to the same juvenile 30 min after the initial exposure display little investigatory behavior. Adult rats were housed individually in polypropylene cages during 3 days of habituation to their new environment. All juveniles were isolated in individual cages for 20 min prior to the beginning of the experiment. The social recognition test consisted of two successive presentations (5 min each) separated by a short period of time where a juvenile rat was placed in the home cage of the adult rat and the time spent by the adult in investigating the juvenile (nosing, sniffing, grooming or pawing) was recorded. At the end of the first presentation, the juvenile was removed and kept in an individual cage during the delay period and re-exposed to the adult rat after 30 min. The first and second presentations were analyzed and then, determined the Ratio of Investigation Duration (RID) which corresponds to the ratio between the time of the second and first expositions.^72,73^

#### 4.4.3 Modified Forced Swim Test

The procedure was a modification of the method proposed previously.^74,75^ The test was conducted in two sessions. In the training session, the rats were placed for 15 min in a tank (25 cm diameter, 60 cm height) that contained water at a temperature of 24 ± 1 °C and depth of 30 cm. Twenty-4 h after the training session, the rats were subjected to the forced swim test (FST) for 5 min, which was video-recorded for subsequent quantification of the following parameters: immobility (i.e., the lack of motion of the entire body, with the exception of only small movements necessary to keep the animal’s head above the water), climbing (i.e., vigorous movements of the forepaws in and out of the water, usually directed against the wall of the tank), and swimming (i.e., large forepaw movements that displaced water and moved the animal’s body around the cylinder, which were more than necessary to keep the head above the water). The water was changed after each animal to avoid the influence of temperature and substances left by previous rats.^27,32^ This test was performed 29 days after the last neurotoxin rotenone injection to allow a complete recovery from hypolocomotion.

### 4.5 Brain Collection and Preparation

For the immunohistochemical studies, rats were deeply anesthetized with a mixture of ketamine (100 mg/kg) and xylazine (20 mg/kg) immediately after the behavioral test and intracardially perfused with saline solution (0.9% NaCl) containing heparin (25000 IU/mL solution), followed by fixative solution (4% of formaldehyde solution in 0.1 M phosphate buffer, pH 7.4). The brains were removed from the skull, postfixed in the fixative solution for 24 h at 4 °C. The brains were then immersed in a 30% sucrose solution (pH 7.4) until they sank, and quickly frozen on dry ice and stored in freezer at −80 °C before sectioning. Coronal series of 30-μm thick sections were cut with a cryostat (Leica CM1850 model, Leica Biosystems, Nussloch, Germany) throughout the rostro-caudal extent of the brain including the following structures (in mm, from bregma): substantia nigra (−4.68 to −5.40) and raphe nuclei (−6.96 to −8.52).^76^

### 4.6 Immunohistochemistry for Tyrosine Hydroxylase (TH) and Tryptophan Hydroxylase (TPH)

For the purpose of TH immunohistochemistry, tissue sections were incubated with primary anti-TH antibody raised in mouse (1:2000; clone LNC1; cat no. MAB318, MilliporeSigma, Darmstadt, Germany), and diluted in phosphate-buffered saline (PBS) that contained 0.15% Triton X-100 (1:500; cat no. AB152, Chemicon, Temecula, CA) overnight at 4 °C. The slides were then incubated with the biotin-conjugated goat antimouse IgG secondary antibody (1:300, cat no. B0529, Sigma, St. Louis, MO) for 2 h at room temperature. After several washes in PBS, the antibody complex was detected using a modification of the ABC system (cat no. PK6101, Vectastain ABC Elite kit, Vector Laboratories, Burlingame, CA), followed by reacting with 3,3′-diaminobenzidine.

For the purpose of TPH immunohistochemistry, tissue sections were incubated with primary anti-TPH antibody raised in sheep (1:1000; cat no. AB1541, MilliporeSigma, Darmstadt, Germany), and diluted in phosphate-buffered saline (PBS) that contained 0.15% Triton X- 100 (1:500; cat no. AB152, Chemicon, Temecula, CA) overnight at 4 °C. The slides were then incubated with the rabbit antisheep IgG secondary antibody (1:400, cat no. ab102297, Abcam, Cambridge, UK) for 2 h at room temperature. After several washes in PBS, the antibody complex was detected using a modification of the ABC system (cat no. PK6101, Vectastain ABC Elite kit, Vector Laboratories, Burlingame, CA), followed by reacting with 3,3′-diaminobenzidine. Afterward, the sections were placed on gelatin-coated slides, let to dry, and then, dehydrated in ascending ethanol concentrations, cleared in xylene and coverslipped.

### 4.7 Unbiased Stereological Quantification of Immunopositive TH and TPH Neurons

TH-immunoreactive (TH-ir) neurons and TPH-ir neurons were stereologically quantified by making use of the image processing program ImageJ (National Institutes of Health – NIH, MD). The mean number of TH-ir and TPH-ir neurons in each hemisphere, obtained through the quantification of 6 and 5 alternated slices, was considered to be representative of the SNpc and raphe nuclei neuronal cells in each animal, respectively. The selected areas were digitized with the digital camera Axiocam ERc 5s attached to the Zeiss Axio Lab.A1 microscope (Zeiss, Germany).

For the quantification of serotonergic neurons, the numbers of TPH-ir neurons were counted in different regions of the DRN at multiple rostrocaudal levels (−7.20, −7.80, −8.04, and −8.52 mm Bregma). The subregions of the DRN analyzed included the dorsal raphe nucleus, dorsal part (DRD) and dorsal raphe nucleus, ventral part (DRV) at −7.20 mm Bregma; the DRD, DRV and dorsal raphe nucleus, ventrolateral part (DRVL) at −7.80 mm Bregma; the DRD, DRV, DRVL and dorsal raphe nucleus, interfascicular part (DRI) at −8.04 mm Bregma; and the dorsal raphe nucleus, caudal part (DRC) and DRI at −8.52 mm Bregma. The quantification of TPH-ir neurons in MRN was performed at −7.80 mm Bregma.

### 4.8 Determination of Dopamine, Noradrenaline, Serotonin, and Metabolites Concentrations

The striatum, amygdala, hippocampus, and prefrontal cortex structures were dissected and stored at −80 °C until the neurochemical quantification. The endogenous concentrations of DA, DOPAC, HVA, NA, 5-HT and 5-HIAA were assayed by reverse-phase high performance liquid chromatography (HPLC) with electrochemical detection (ED). Briefly, the system consisted of a Synergi Fusion-RP C-18 reverse-phase column (150 × 4.6 mm i.d., 4 m particle size) fitted with a 4 × 3.0 mm precolumn (Security Guard Cartridges Fusion-RP); an electrochemical detector (ESA Coulochem Electrochemical Detector) equipped with a guard cell (ESA 5020) with the electrode set at 350 mV and a dual electrode analytical cell (ESA 5011A); a LC-20AT pump (Shimadzu, Columbia, MD) equipped with a manual Rheodyne 7725 injector with a 20 μL loop. The column was maintained inside in a temperature-controlled oven (25 °C; Shimadzu). The cell contained two chambers in series: each chamber including a porous graphite coulometric electrode, a double counter electrode and a double reference electrode. Oxidizing potentials were set at 100 mV for the first electrode and at 450 mV for the second electrode. The tissue samples were homogenized with an ultrasonic cell disrupter (Sonics, Newtown, CT) in 0.1 M perchloric acid containing sodium metabisulfite 0.02% and internal standard. After centrifugation at 10,000*g* for 30 min at 4 °C, 20 μL of the supernatant was injected into the chromatograph. The mobile phase, used at a flow rate of 1 mL/min, had the following composition: 20 g citric acid monohydrated (Merck, Darmstadt, Germany), 200 mg octane-1-sulfonic acid sodium salt (Merck, Darmstadt, Germany), 40 mg ethylenediaminetetraacetic acid (EDTA) (Sigma, St. Louis, MO), 900 mL HPLC-grade water. The pH of the buffer running solution was adjusted to 4.0 then filtered through a 0.45 m filter. Methanol (Merck, Darmstadt, Germany) was added to give a final composition of 10% methanol (v/v). The neurotransmitters and metabolites concentrations were calculated using standard curves that were generated by determining in triplicate the ratios between three different known amounts of the internal standard. The unit was expressed as ng/g of wet weight.

### 4.9 Statistical Analysis

Numerical data were tested for normality (D’Agostino-Pearson omnibus’ test) and equal variance (standard unpaired *t* test for unequal variance). Data were normalized when they did not pass the normality test. Homocedasticity between groups was assessed with the Bartlett test and normal distribution of the data with the Kolmogorov–Smirnov test. The data from the open field test, and investigation duration in the social recognition test were analyzed using two-way analysis of variance (ANOVA), followed by the Bonferroni post hoc test for multiple comparisons. The social recognition task (RID), the forced swim test, and neurochemistry data were analyzed using Student’s unpaired *t*-test. The results obtained from immunohistochemistry were analyzed using one-way ANOVA, followed by Tukey post hoc test. The values were expressed as mean ± standard error of the mean (S.E.M.). Differences were considered significant if *p* < 0.05. Calculations were made with the GraphPad Prism for Windows software (version 6.01; GraphPad Software, San Diego).

## 5 Conclusion

In summary, the present study demonstrated that the systemic administration of rotenone induced short-term memory impairment and depressive-like behavior. Moreover, the rotenone exposure led to distinct effects in the serotonergic system, indicated by a decrease in serotonergic neurons of the MRN and a reduction in 5-HT levels in the prefrontal cortex and striatum. Likewise, the DA and NA levels were found reduced in the prefrontal cortex, striatum, and amygdala.

The involvement of the neurotransmitters 5-HT, DA, and NA is probably necessary for the development of nonmotor disturbances in the animal model of PD induced by rotenone, which appear to parallel clinical data.

## Supplementary Material



## Notes

Additional graphs and representative photomicrographs illustrating the effect of rotenone administration on serotonergic neurons of DRN labeled for TPH evaluated 24 h and 28 days after the last injection (PDF)
