# Supplementary material for: Involvement of Raphe Nuclei in Depressive-like Behaviors and Short-Term Memory in an Animal Model of Parkinson’s Disease
Source: ACS Chem Neurosci. 2026 Jun 25;17(14):2628–40. doi: 10.1021/acschemneuro.5c00987 (PMC13377602; doi:10.1021/acschemneuro.5c00987)
Supplement: Supplementary file 1 [file cn5c00987_si_001.pdf]

Supplemental material

Fig. S1

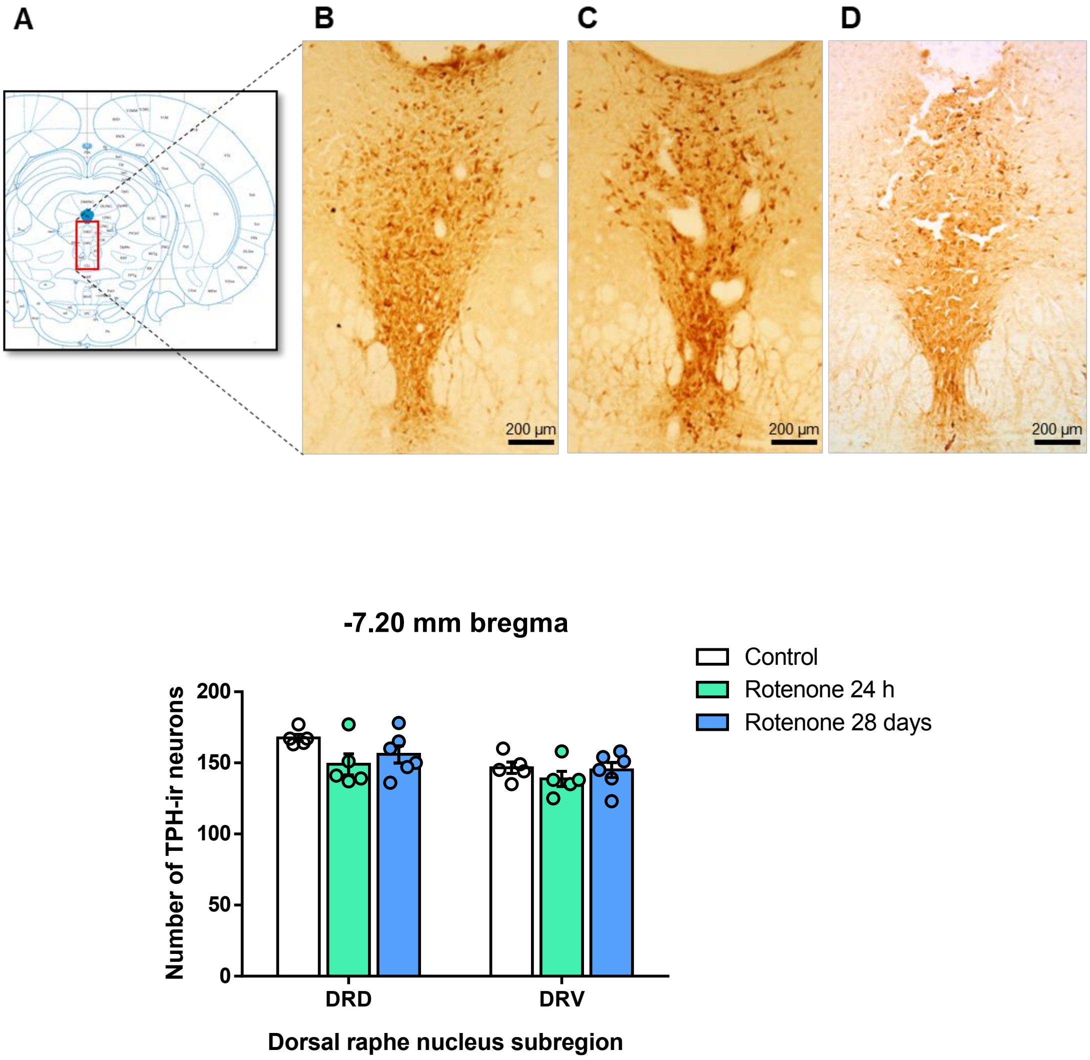

The effect of rotenone administration on serotonergic neurons of DRN labeled for TPH evaluated 24 h and 28 days after the last injection. (A) Clipping of the Paxinos & Watson atlas indicating the DRN sub-regions analyzed according to the coordinate -7.20 mm. (B) Representative photomicrography of TPH-ir neurons of rostral DRN in the control group. (C) Rotenone group 24 h. (D) Rotenone group 28 days. (E) Stereological quantification of the total number of TPH-ir neurons in the DRN sub-regions for each group. The values are expressed as mean ± SEM (n = 5-6/group) and analyzed by one way ANOVA followed by Tukey's *post hoc* test). DRN: dorsal raphe nucleus; DRD: DRN part or dorsal

sub-region; DRV: DRN ventral part; TPH-ir: tryptophan hydroxylase immunoreactive.

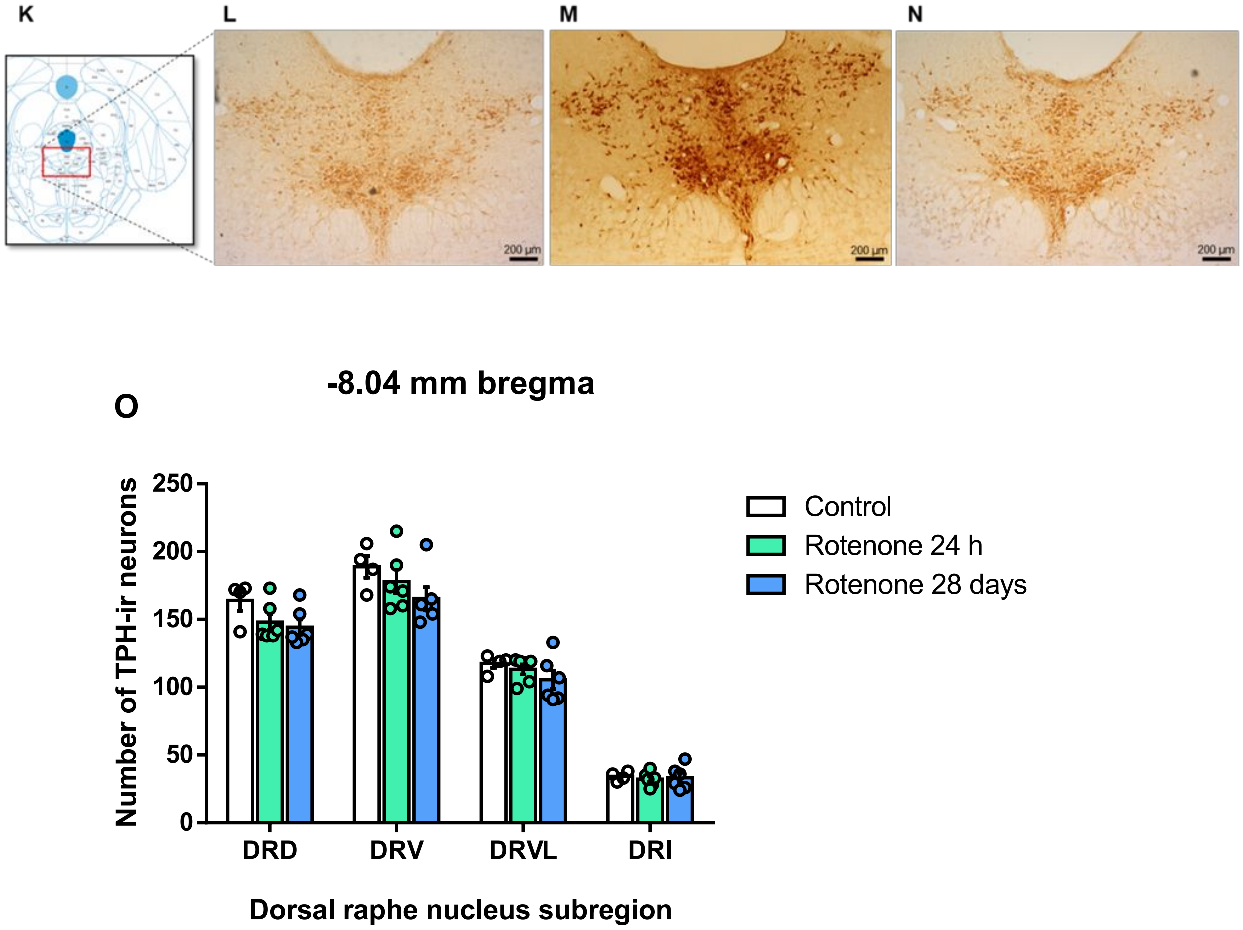

The effect of rotenone administration on serotonergic neurons of DRN labeled for TPH evaluated 24 h and 28 days after the last injection. (K) Clipping of the Paxinos & Watson atlas indicating the DRN sub-regions analyzed according to the coordinate -8.04 mm. (L) Representative photomicrography of medial DRN TPH-ir neurons in the control group. (M) Rotenone group 24 h. (N) Rotenone group 28 days. (O) Stereological quantification of the total number of TPH-ir neurons in the DRN sub-regions for each group. The values are expressed as mean ± SEM (n = 4-6/group) and analyzed by one way ANOVA followed by Tukey's *post hoc* test). DRN: dorsal raphe nucleus; DRD: DRN dorsal part; DRV: DRN ventral part; DRVL: DRN ventrolateral part; DRI: DRN part interfascicular; TPH-ir: tryptophan hydroxylase immunoreactive.

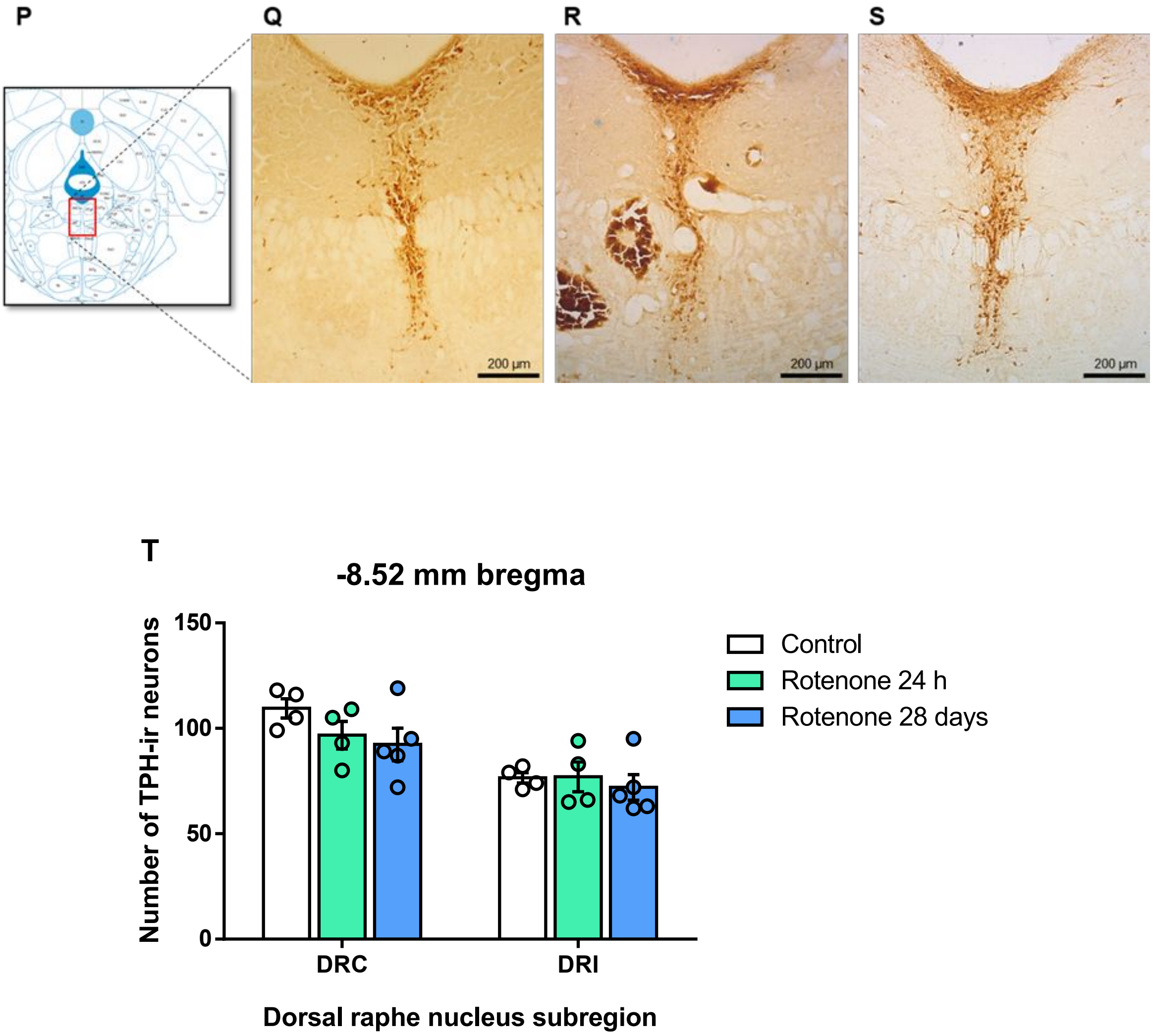

The effect of rotenone administration on serotonergic neurons of DRN labeled for TPH evaluated 24 h and 28 days after the last injection. (P) Clipping of the Paxinos & Watson atlas indicating the DRN sub-regions analyzed according to the coordinate -8.52 mm. (Q) Representative photomicrography of caudal DRN TPH-ir neurons in the control group. (R) Rotenone group 24 h. (S) Rotenone group 28 days. (T) Stereological quantification of the total number of TPH-ir neurons in the DRN sub-regions for each group. The values are expressed as mean ± SEM (n = 4-5/group) and analyzed by one way ANOVA followed by Tukey's *post hoc* test. DRN: dorsal raphe nucleus; DRI: DRN interfascicular part; DRC: DRN caudal part; TPH-ir: tryptophan hydroxylase immunoreactive.
